# A prospective cohort study of postoperative complications in the management of perforated peptic ulcer

**DOI:** 10.1186/1471-2482-6-8

**Published:** 2006-06-16

**Authors:** Smita S Sharma, Manju R Mamtani, Mamta S Sharma, Hemant Kulkarni

**Affiliations:** 1 Department of Surgery, Indira Gandhi Government Medical College, Nagpur, India; 2 Lata Medical Research Foundation, Nagpur, India; 3 Department of Physiology, Indira Gandhi Government Medical College, Nagpur, India

## Abstract

**Background:**

With dwindling rates of postoperative mortality in perforated peptic ulcer that is attributable to H_2_-receptor blocker usage, there is a need to shift the focus towards the prevention of postoperative morbidity. Further, the simultaneous contribution of several putative clinical predictors to this postoperative morbidity is not fully appreciated. Our objective was to assess the predictors of the risk, rate and number of postoperative complications in surgically treated patients of perforated peptic ulcer.

**Methods:**

In a prospective cohort study of 96 subjects presenting as perforated peptic ulcer and treated using Graham's omentoplatsy patch or gastrojejunostomy (with total truncal vagotomy), we assessed the association of clinical predictors with three domains of postoperative complications: the risk of developing a complication, the rate of developing the first complication and the risk of developing higher number of complications. We used multiple regression methods – logistic regression, Cox proportional hazards regression and Poisson regression, respectively – to examine the association of the predictors with these three domains.

**Results:**

We observed that the risk of developing a postoperative complication was significantly influenced by the presence of a concomitant medical illness [odds ratio (OR) = 8.9, p = 0.001], abdominal distension (3.8, 0.048) and a need of blood transfusion (OR = 8.2, p = 0.027). Using Poisson regression, it was observed that the risk for a higher number of complications was influenced by the same three factors [relative risk (RR) = 2.6, p = 0.015; RR = 4.6, p < 0.001; and RR = 2.4, p = 0.002; respectively]. However, the rate of development of complications was influenced by a history suggestive of shock [relative hazards (RH) = 3.4, p = 0.002] and A^- ^blood group (RH = 4.7, p = 0.04).

**Conclusion:**

Abdominal distension, presence of a concomitant medical illness and a history suggestive of shock at the time of admission warrant a closer and alacritous postoperative management in patients of perforated peptic ulcer.

## Background

Surgical emergency due to a perforated peptic ulcer – whether treated laparoscopically or by open repair – is associated with a significant postoperative morbidity and mortality [1,2]. Therefore, risk-stratification of these subjects provides surgeons with an important tool to plan the management. However, a generalized use of the currently popular risk-stratification strategies in patients of perforated peptic ulcer suffers from one or more of the following three limitations. First, most of the available strategies are better predictors of postoperative mortality than morbidity [3]. Nonetheless, the use of H_2_-receptor blockers has significantly reduced the postoperative mortality [4,5]. Consequently, it is now imperative and appropriate to recognize the determinants of the postoperative morbidity. Second, the studies [3,6-14] that assess the association of clinical predictors with postoperative morbidity have examined only one domain of postoperative complication – the risk of developing a complication. There exist subtly distinct but additional other domains of the complications namely the rate of development of a complication and the number of complications that simultaneously or sequentially develop in a given patient. Arguably, the predictors that influence the risk of developing a complication may be different from the ones that can potentially influence these other domains of complications. Third, the isolated effect of a predictor is unlikely to be the same as the concurrent and concomitant effect of the same predictor in a multiple regression context with potentially better model fits resulting from multiple rather than single variable analyses [15]. Considering these three critical issues, we conducted a prospective cohort study of the determinants of postoperative complications in subjects presenting as perforated peptic ulcer.

## Methods

### Study protocol

This prospective cohort study of patients with perforated peptic ulcer under care of all the surgical units at Indira Gandhi Medical College and Hospital Nagpur, was carried out between January 2000 and July 2001. All of the patients were admitted to the study center as surgical emergencies. Subjects who presented with signs and symptoms suggestive of perforated peptic ulcer and who acceded to a fully informed consent were included in the study. As preoperative measures the patients were treated with antibiotics, intravenous fluids, Ryle's tube aspiration and blood transfusion when indicated. All the patients underwent plain x-ray of the abdomen in standing position, covering both domes of diaphragm with the purpose of demonstrating free gas under diaphragm. The diagnosis of perforation was made on clinical history, examination and presence of gas under diaphragm but was confirmed only on exploration. Before initiating a surgical intervention however, a fully informed consent was sought from the patient or the next of kin.

All the patients were surgically treated by open repair of the perforation. The abdomen was opened with a midline or paramedian incision, the peritoneal spillage was sucked out and the perforation was located which was closed. After irrigating with at least 3 liters of warm normal saline, the peritoneal cavity was mopped thoroughly and abdomen was closed. The decision to keep a drain was based on the degree of peritoneal spillage which was estimated by measuring the amount of fluid in the suction bottle aspirated from opening the peritoneum till the stage of peritoneal lavage. All patients received postoperative intravenous fluids, and Ryle's tube aspiration till return of intestinal motility. Postoperative complications were noted along with their time of onset since operation. Surviving patients were discharged in a stable condition.

### Predictors and outcomes

Our study assessed the association of 17 factors that could potentially influence the postoperative morbidity and mortality – 14 measured on admission and 3 measured operatively. The predictors measured on admission were age, sex, duration of pain, vomiting, abdominal distension, history suggestive of oliguria, history suggestive of acid peptic disease, history suggestive of shock, history suggestive of dehydration, history of smoking, presence of associated medical condition(s), tenderness, presence of bowel sounds and blood group. All the variables related to history were ascertained during the complete clinical workup by SSS and were measured by interrogating the patient or the next of kin. Except for the history of smoking and history suggestive of acid peptic disease, all other variables related to history within the past three days. Specifically, history suggestive of shock was defined as symptoms of increased respiratory rate, cyanosis and altered state of consciousness in addition to a history suggestive of oliguria. The predictors measured during operation were the amount of peritoneal spillage, the site of perforation and the size of perforation.

The postoperative complications that we specifically looked for were wound infection, burst abdomen, hematemesis, gastro-duodenal fistula, enterocutaneous fistula, intraperitoneal abscess, respiratory complications and death. We assessed the influence of the predictor variables on three outcomes – the risk of developing a complication, the rate of complication development and the number of complications that developed.

### Statistical analysis

Our primary objective was to assess the influence of the predictor variables on the postoperative complications in a multiple regression environment. Since the number of postoperative complication events in the present study limited the number of statistical comparisons between predictors and each type of the developing complication, we created a composite variable which we dubbed "postoperative complication". This variable was coded as 1 if any of the complications listed above were noted during the follow-up and 0 otherwise. We first examined the influence of the predictors on the rate at which a postoperative complication developed – first in a univariate fashion using Kaplan-Meier plots and logrank test and then in a multiple regression context using stepwise Cox proportional hazards regression analyses. The validity of the Cox proportional hazards model was assessed using the Schoenfeld residuals. The following Cox proportional hazards model was used to assess the influence of the covariates on the rate of developing a complication:

λ(t|z)=λ0teβTz
 MathType@MTEF@5@5@+=feaafiart1ev1aaatCvAUfKttLearuWrP9MDH5MBPbIqV92AaeXatLxBI9gBaebbnrfifHhDYfgasaacH8akY=wiFfYdH8Gipec8Eeeu0xXdbba9frFj0=OqFfea0dXdd9vqai=hGuQ8kuc9pgc9s8qqaq=dirpe0xb9q8qiLsFr0=vr0=vr0dc8meaabaqaciaacaGaaeqabaqabeGadaaakeaacqaH7oaBdaqadaqaaiabdsha0jabcYha8jabdQha6bGaayjkaiaawMcaaiabg2da9iabeU7aSnaaBaaaleaacqaIWaamaeqaaOGaemiDaqNaemyzau2aaWbaaSqabeaacqaHYoGydaahaaadbeqaaiabdsfaubaaliabdQha6baaaaa@3FAE@ where, λ(t|z) represents the hazard conditional on the baseline hazard λ_0_, t represents the time, **β**^**T **^is the transposed matrix of regression coefficients and **z **represents the matrix of covariates. A logarithmic transformation of this equation yields the relative hazards for each covariate based its corresponding regression coefficient.

We also assessed the association of each predictor with risk of developing a postoperative complication using stepwise multiple unconditional logistic regression analyses. In these analyses, we defined the odds of developing a complication in the following way:

Pr⁡(c|z)=eβTz1+eβTz
 MathType@MTEF@5@5@+=feaafiart1ev1aaatCvAUfKttLearuWrP9MDH5MBPbIqV92AaeXatLxBI9gBamXvP5wqSXMqHnxAJn0BKvguHDwzZbqegyvzYrwyUfgarqqtubsr4rNCHbGeaGqiA8vkIkVAFgIELiFeLkFeLk=iY=Hhbbf9v8qqaqFr0xc9pk0xbba9q8WqFfeaY=biLkVcLq=JHqVepeea0=as0db9vqpepesP0xe9Fve9Fve9GapdbaqaaeGacaGaaiaabeqaamqadiabaaGcbaGagiiuaaLaeiOCaiNaeiikaGIaem4yamMaeyiFaWNaemOEaONaeiykaKIaeyypa0ZaaSaaaeaacqWGLbqzdaahaaWcbeqaaiabek7aInaaCaaameqabaGaemivaqfaaSGaemOEaOhaaaGcbaGaeGymaeJaey4kaSIaemyzau2aaWbaaSqabeaacqaHYoGydaahaaadbeqaaiabdsfaubaaliabdQha6baaaaaaaa@5485@ where, c represents a postoperative complication and **β**^**T **^and **z **have same meaning as described above in the context of the Cox regression model. Again, a logarithmic transformation of this equation yields the odds ratios for each covariate.

We then created another outcome variable that counted the number of postoperative complications noted in each study subject. To assess the influence of the predictors on this variable we used stepwise Poisson regression analysis since this regression analysis is used for outcome variables containing count data. The following regression equation was used for the Poisson regression analysis: log (#c) = β_0 _+ β_1_z_1 _+ β_2_z_2 _+ ... + β_n_z_n_, where, #c represents the number of complications, βs represent the regression coefficients and zs represent the covariates. All the stepwise regression procedures we used were implemented using a backward elimination approach with a retaining criterion of 0.2. The significance of association was assessed at an α-error rate of 0.05. The statistical analyses were conducted using Stata 8 (Stata Corp, College Station, Texas) software package.

## Results

### Characteristics of study subjects

**Figure 1:**
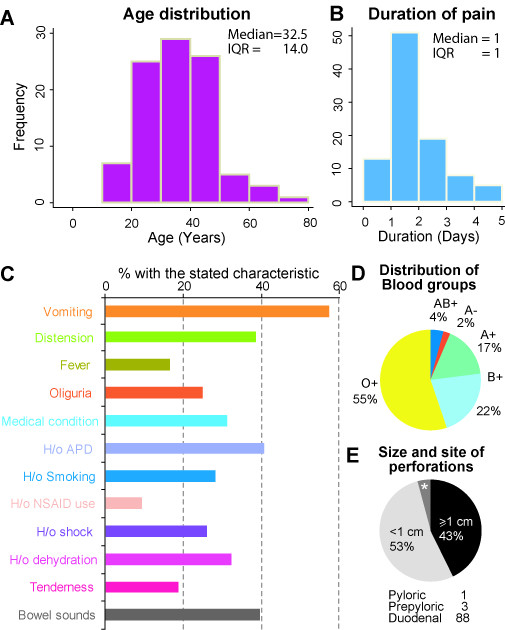
**Characteristics of the study subjects**. **(A and B) **Histograms showing the distribution of age (A) and duration of pain (B) in the study subjects. IQR, interquartile range. **(C) **Bar chart showing the proportion of study subjects with the indicated characteristics on admission. **(D and E) **Pie chart showing the distribution of blood groups (D) and size of perforation in the study subjects (E). At the bottom of panel E is given the distribution of the site of perforation. * represents the four subjects in which the site of perforation could not be determined intra-opeartively.

We recruited a total of 96 subjects. The characteristics of these subjects with regard to the study variables are shown in Figure 1. The subjects – on an average – were relatively young (Figure 1A) with a very high male:female ratio (18.2:1) and captured rather early in the disease process as ~67% had pain for one day or less (Figure 1B). In the order of frequency the most common characteristics on admission (as shown in Figure 1C) were vomiting (57%), history suggestive of acid peptic disease (41%), presence of bowel sounds (40%), abdominal distension (39%), signs of dehydration (32%), associated medical condition (31%), history of smoking (28%), history of shock (26%), oliguria (25%), generalized abdominal tenderness (19%) and fever (17%). The associated medical conditions included chronic obstructive airway disease (COAD, 10 cases), ischemic heart disease (IHD, 10 cases), essential hypertension (HT, 7 cases) type 2 diabetes (DM, 6 cases), pulmonary tuberculosis (PTB, 3 cases) and human immunodeficiency virus (HIV) infection (3 cases). We also noted (Figure 1D) that the ORh^+ ^blood group was most common (55%) followed by B^+ ^(22%), A^+ ^(17%), AB^+ ^(4%) and A^- ^(2%).

During the emergency open repair, we observed that the amount of peritoneal spillage varied widely from absent to as large as 3 liters. The median (interquartile range, IQR) amount of this contaminating fluid was 125 ml (950 ml). In the study subjects we could locate a perforation in 92 (96%) subjects. In all cases the peritoneal fluid spillage was sterile. In the remaining four subjects therefore no surgical repair could be carried out – 2 subjects were treated with drain insertion and H_2_-receptor antagonists while 2 were treated only with H_2_-receptor antagonists postoperatively. In the 92 subjects in whom the perforation was operatively confirmed, 37 (40%) had a large perforation (maximum diameter ≥1 cm) while 55 subjects (60%) had a small perforation (Figure 1E). None of these 92 subjects had multiple perforations. Eighty eight of these subjects had the perforation located in the first part of duodenum, 1 had the perforation in the pyloric region while 3 had it in the prepyloric region. In 87 (94%) subjects the perforation was closed with Graham's omentoplasty patch [16] while in the remaining five subjects we used gastrojejunostomy with total truncal vagotomy [17].

### Postoperative complications

During their hospital stay, a total of 29 (30%) study subjects developed a total of 50 events of postoperative complications that included one or more of the following: cutaneous wound infection (16); respiratory complications (15) including pneumonitis (10) and acute exacerbation of COAD (5); wound dehiscence necessitating the use of a tension suture (6); death (5); and postoperative fistula and burst abdomen (4 each). In the subjects who died the causes of death were sepsis (three cases); pneumonitis and respiratory acidosis (one case); and type 2 diabetes leading to ketoacidosis (one case). In the eight subjects with cutaneous wound infections in whom the culture was positive for bacteria there were four subjects with *Staphycoccus aureus *infection, three with *Klebsiella sp *and one with *Pseudomonas aeruginosa*. In all, 16 subjects developed only one complication, 7 subjects developed 2 complications each, 4 developed 3 complications each and 2 developed 4 complications each. Again, using a multiple linear regression model, we observed that each additional complication prolonged the hospital stay of the patients by 1.25 days (95% confidence interval 0.45 – 2.05 days, p = 0.002) over the average hospital stay of 8.85 days (95% confidence interval 8.25 – 9.45 days) in subjects who did not develop a complication.

From a surgical standpoint, 22 (75.9%) of the subjects (a total of 35 complication events) developing a postoperative complication, needed a surgical intervention. All cases of wound infection were subjected to closure by secondary intension while the cases of wound dehiscence and burst abdomen were treated by exploratory peritoneal lavage followed by a tension suture. For placing the tension suture, an Ethilon suture was first used from the skin (3 cm distant from the edge of the wound) and the peritoneum including all tissue layers followed by a mass closure using prolene suture. Three out of the four subjects who developed post-operative enterocutaneous fistula were managed by re-exploration and gastrojejunostomy while one case of post-operative fistula died of sepsis before a re-exploration could be undertaken.

### Clinical predictors of postoperative complications: single variable analyses

**Figure 2:**
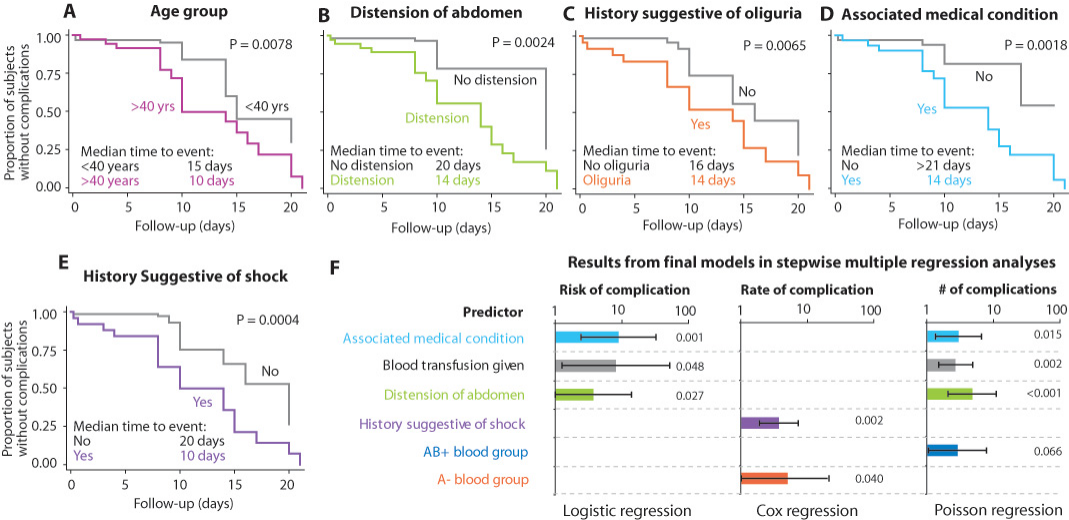
**Association of the study factors with postoperative complications**. **(A to E) **Kaplan-Meier (K-M) plots showing the time to development of postoperative complication based on five significant study factors. The significance values (P) was estimated using logrank test. The median time to a postoperative complication across the two categories of each predictor is shown inside each K-M plot. The colors used in the plots correspond to the colors used figure 1A and 1C. The remaining K-M plots are shown in part A of the Additional file. **(F) **Multiple regression analyses assessing the association of the study factors with three outcomes related to postoperative complications – risk of developing complication (left), rate of developing complication (center) and number of complications (right). For each of these outcomes, we used appropriate stepwise multiple regression models (indicated at the bottom and described in Methods section). The bars represent the risk estimate and the error bars represent the 95% confidence interval. The numbers opposite the bars are significance values obtained from the respective regression analyses. The full results of these models are given in part B of the Additional File 1

We first set out to examine which of the study predictors – considered singly – influenced the rate at which the study subjects developed postoperative complications. For this purpose, we constructed a series of Kaplan-Meier plots and tested the corresponding statistical significance using logrank tests. All the Kaplan-Meier plots are shown in the supplementary information (Additional file 1, Part A). For these analyses we categorized the continuous variables. Age was categorized into three groups based on tertiles and since the lower and middle groups did not differ in terms of the rate of progression to complications, we compared the upper tertile (40 years) with a combination of the lower and middle tertile (Figure 2A). Duration of pain was categorized as <24 hours, 24–48 hours, 48–72 hours and more than 72 hours; the size of perforation was dichotomized as less than 1 cm and ≥1 cm while the amount of peritoneal spillage was dichotomized as <1 L and ≥1 L. The univariate analyses (Figure 2A to 2E and Additional File 1 Part A) showed that age > 40 years (p = 0.0078), vomiting (p = 0.0125), abdominal distension (0.0024), fever (p = 0.0133), oliguria (p = 0.0065), associated medical condition (p = 0.0018), history suggestive of shock (p = 0.0004), dehydration (p = 0.0018) and peritoneal spillage (p = 0.0079) significantly influenced the rate of postoperative complication.

### Clinical predictors of postoperative complications: multiple regression analyses

Figure 2F summarizes the results of multiple regression analyses while Part B of additional file 1 details these results. We observed that presence of a concomitant medical illness; distension of abdomen on admission and the need for immediate postoperative blood transfusion were significant predictors of the risk developing a postoperative complication. In contrast, the rate at which a postoperative complication developed was significantly dictated by the history suggestive of shock on admission and the possession of the A^- ^blood group. However, the risk of developing a higher number of complications was significantly dictated by AB^+ ^blood group in addition to the three factors that were significantly associated with the risk of developing a postoperative complication. This greatly reduced set of predictors that was retained as significant after the multiple regression analyses indicated that several predictors might be capturing overlapping prognostic information. This conjecture was fully supported by the full correlation matrix among all the predictors (shown in Part C of the Additional file1 ).

## Discussion

In this study, we addressed the issue of postoperative complications in cases of perforated peptic ulcer from three perspectives: "whether" the predictors prognosticate the likelihood of developing a complication; "how rapidly" do the postoperative complications develop across different categories of the predictors and "how many" events of postoperative complications develop across the categories of the predicting variables. Our reasoning for these three dimensions of postoperative complications was based on the premise that together these three dimensions will provide a holistic view of the concept of postoperative complications. The first of these dimensions has been a focus of previous studies [2] but the second and third dimensions are novel to this study and attempt to capture the surgeon's need for quicker actions and the need to be aware of the severity of complications, respectively. To assess whether our premise is rational, we conducted some further analyses. Using a Poisson regression analysis, we assessed if a higher number of postoperative complications increased the risk of mortality. It did (OR for death for each additional postoperative complication = 2.14, 95% CI 1.21 – 3.82, P = 0.009), suggesting that higher number of complications was also an important outcome to measure.

Our results reaffirm that concomitant medical illness and distension of abdomen are simple and accurate predictors of postoperative complications in surgically treated patients of perforated peptic ulcer. These predictors are clinically relevant since both can be measured at the time of admission. While presence of concomitant medical illness has been previously identified as a significant predictor of the risk of postoperative morbidity and mortality by several authors [8,10,12,13]; to our knowledge the finding that abdominal distension also strongly predicts the risk and number of postoperative complications has not been previously reported. For the purpose of our study, we defined abdominal distension as any visible abdominal bloating. In a large Italian study [18] assessing 9,883 dyspeptic subjects with discomfort as the predominant symptom, moderate to severe abdominal distension was observed in ~37% of subjects which concurs with the proportion of subjects with distension observed in our study. In patients of perforated peptic ulcer, presence of abdominal distension can indicate the amount of peritoneal spillage. Indeed, in our study subjects the mean amount of peritoneal spillage was significantly higher (Mann-Whitney test p < 3 × 10^-7^) in subjects with distension (1.19 L) than without (0.32 L). Thus, in our study we identified abdominal distension to be a statistically, biologically and clinically meaningful predictor of the risk and number of postoperative complications. More importantly, considered in a multiple variable context the strong association between abdominal distension and postoperative complications masked the univariate association of the amount of peritoneal spillage with the same outcome underscoring the importance of multiple regression methods of data analysis.

Even though we did observe that the size of perforation was a strong predictor of the amount of peritoneal spillage (data not shown), the size was not associated with risk, rate or number of postoperative complications either in single or multiple variable contexts. A recent study [19] has suggested that size of perforation should be used in risk-stratification. However, we could not replicate their findings in our study. Therefore, use of size of perforation as a predictor of postoperative morbidity and mortality may require further validation in larger studies before it can be accepted for general use. In any case, size of perforation is an intra-operative finding and therefore its use as a predictor of postoperative complication would rank slightly below the simple and early predictors like concomitant medical illness and abdominal distension. This argument also applies to the significant association of the blood transfusion with postoperative complications that we observed in our study. However, our study shows that subjects with a history suggestive of shock at the time of admission may need a more alacritous postoperative management since the postoperative complications are likely to develop much faster in these subjects. In these regards, our findings agree with those of Testini et al [20].

Before generalizing the results of our study several caveats need to be mentioned. First, it is possible that there is a loss of useful clinical information subsequent to the strategy of combining all the different types of postoperative complications into a single conglomerate variable. In theory, we could have used one of the two following approaches for the analysis of multiple complications. We could either have used multiple regression models for each outcome variable or we could have used novel multivariate analytical techniques like multivariate Cox models [21], Generalized Estimating Equations [22] or Structural Equations Modeling [23]. While this is true, we used the composite variable "postoperative complication" for the following three practical reasons: i) clinically, it is needed to guard against all types of complications rather than any one type of complication; ii) statistically, there is an advantage in combining the events into a composite variable since it is likely to provide the necessary statistical power; iii) literature-wise, there are several examples [24-28] of this line of analysis to assess the predictors of postoperative complications.

Second, all the subjects in the present study underwent open repair. On the contrary, the current surgical approach in treating perforated peptic ulcers is tilting strongly in favour of the laparoscopic repair [1,2,29]. The type and incidence of postoperative complications observed in the present study may not fully represent those after laparoscopic repair. Third – and in the same vein – most of the study subjects underwent a single type of surgical repair. Thus, these results will mostly be applicable to open repair with Graham's omentoplasty patch. Fourth, our study could not identify age as a significant predictor probably since our study sample contained only four subjects aged 60 or above – a factor usually considered to be a strong predictor of the risk of postoperative complication [6,10,11,13,14]. Fifth, the association of blood groups with postoperative complications was only modest as indicated by the wide confidence intervals around point estimates of the risk measures and may mirror the difficulty in procuring blood for transfusion immediately post-surgery in subjects with rare blood groups. Sixth, we did not study the association of *H. pylori *with the postoperative outcomes because of lack of necessary facilities at the study center. Absence of this important potential factor [30,31] in the list of statistically significant predictors does not indicate absence of a possible lack of an association of *H. pylori *with postoperative complications. Seventh, since our duration of postoperative follow up was relatively short, we could not estimate the incidence and risk factors of potential re-leak after the surgery. Lastly, some of the risk factors we identified are a part of existing scoring systems like the Boey score.[32] However, the purpose of the present investigation was to focus on the individual components rather than composite risk scoring systems.

## Conclusion

In the light of the abovementioned caveats, our study demonstrates that abdominal distension, presence of concomitant medical illness and history suggestive of shock are the three major and early clinical predictors of the risk, rate and/or number of postoperative complications in patients with perforated peptic ulcer.

## Competing interests

The author(s) declare that they have no competing interests.

## Authors' contributions

SSS conceptualized the study and conducted the surgical management of study subjects.

MRM was involved in the conceptualization of the study. She also conducted parts of statistical analyses and created the artwork.

MSS contributed to a critical review of the manuscript.

HK conducted parts of statistical analysis and wrote the manuscript.

All authors reviewed and approved the manuscript.

## Pre-publication history

The pre-publication history for this paper can be accessed here:



## Supplementary Material

Additional File 1 **SharmaSupplement1**. Part A of this file contains a series of Kaplan-Meier plots (along with logrank test significance values) showing the univariate influence of the study predictors on the rate of development of postoperative complications. Part B of this file contains the output from Stata software of the final models from multiple regression analyses for the rate of developing postoperative complications, the risk of developing postoperative complications and the number of complications. Lastly, Part C of this file tabulates the correlation matrix for the prognostic predictors studied and their statistical significance. Click here for file
