# Supplementary material for: A prospective cohort study of postoperative complications in the management of perforated peptic ulcer
Source: BMC Surg. 2006 Jun 16;6:8. doi: 10.1186/1471-2482-6-8 (PMC1544354; doi:10.1186/1471-2482-6-8)
Supplement: Additional File 1 — SharmaSupplement1. Part A of this file contains a series of Kaplan-Meier plots (along with logrank test significance values) showing the univariate influence of the study predictors on the rate of development of postoperative complications. Part B of this file contains the output from Stata software of the final models from multiple regression analyses for the rate of developing postoperative complications, the risk of developing postoperative complications and the number of complications. Lastly, Part C of this file tabulates the correlation matrix for the prognostic predictors studied and their statistical significance. [file 1471-2482-6-8-S1.doc]

Additional File 1

# A prospective study of postoperative complications in the management of perforated peptic ulcer

Smita S Sharma, Manju R Mamtani, Mamta S Sharma, Hemant Kulkarni

***Part A***

**Kaplan-Meier plots and logrank test results for the study variables on the rate of developing postoperative complication in cases of perforated peptic ulcer**

Codes:

0 = age <29 years (lower tertile)

1 = age ≥29 – 40 years (middle tertile)

2 = age >40 years (upper tertile)

Logrank p = 0.0222

Codes:

0 = Female

1 = Male

Logrank p = 0.6777

Codes

0 = <24 hours

1 = 24 – <48 hours

2 = 48 – <72 hours

3 = ≥ 72 hours

Logrank p = 0.1068

Logrank p = 0.0125

Logrank p = 0.0024

Logrank p = 0.0133

Logrank p = 0.0065

Codes

0 = No associated medical condition

1 = One or more associated associated condition(s)

Logrank p = 0.0018

Codes

0 = No history suggestive of acid peptic disease

1 = History suggestive of acid peptic disease

Logrank p = 0.3930

Logrank p = 0.8132

Logrank p = 0.6935

Logrank p = 0.0004

Logrank p = 0.0018

Logrank p = 0.5507

Logrank p = 0.1124

Logrank p = 0.1297

Logrank p = 0.0079

Logrank p = 0.2715

Logrank p = 0.0064

Logrank p = 0.1026

***Part B***

**Results of stepwise multiple regression analyses for the three statistical outcomes related to postoperative complications**

**Risk of developing a postoperative complication**

**(Results of stepwise logistic regression analysis)**

begin with full model

p = 0.9956 >= 0.1000 removing vomitting

p = 0.8353 >= 0.1000 removing oliguria

p = 0.7474 >= 0.1000 removing fever

p = 0.5450 >= 0.1000 removing hsodehydration

p = 0.7028 >= 0.1000 removing hsoshock

p = 0.5661 >= 0.1000 removing agecat2

p = 0.4026 >= 0.1000 removing bowelsounds

p = 0.3909 >= 0.1000 removing a_plus

p = 0.3469 >= 0.1000 removing tenderness

p = 0.2678 >= 0.1000 removing ab_plus

p = 0.1795 >= 0.1000 removing b_plus

p = 0.2870 >= 0.1000 removing durationofpaincollapsed

Logistic regression Number of obs = 94

LR chi2(3) = 46.63

Prob > chi2 = 0.0000

Log likelihood = -33.053647 Pseudo R2 = 0.4136

------------------------------------------------------------------------------

adverseevent | Odds Ratio Std. Err. z P>|z| [95% Conf. Interval]

-------------+----------------------------------------------------------------

medcondi | 8.97173 5.955989 3.31 0.001 2.442283 32.95766

distension | 3.772621 2.532016 1.98 0.048 1.012405 14.05827

bloodtrans~n | 8.195624 7.817256 2.21 0.027 1.263799 53.1479

------------------------------------------------------------------------------

**Rate of developing a postoperative complication**

**(Results of stepwise Cox regression analysis)**

begin with full model

p = 0.9735 >= 0.1000 removing bloodtransfusion

p = 0.9133 >= 0.1000 removing a_plus

p = 0.8702 >= 0.1000 removing durationofpaincollapsed

p = 0.8298 >= 0.1000 removing tenderness

p = 0.7131 >= 0.1000 removing vomitting

p = 0.7703 >= 0.1000 removing fever

p = 0.6036 >= 0.1000 removing hsodehydration

p = 0.6218 >= 0.1000 removing agecat2

p = 0.5575 >= 0.1000 removing oliguria

p = 0.5028 >= 0.1000 removing distension

p = 0.2760 >= 0.1000 removing bowelsounds

p = 0.2765 >= 0.1000 removing ab_plus

p = 0.2363 >= 0.1000 removing b_plus

p = 0.1384 >= 0.1000 removing medcondi

Cox regression -- Breslow method for ties

Entry time 0 Number of obs = 96

LR chi2(2) = 12.93

Prob > chi2 = 0.0016

Log likelihood = -89.186269 Pseudo R2 = 0.0676

------------------------------------------------------------------------------

hospitalstay | Haz. Ratio Std. Err. z P>|z| [95% Conf. Interval]

-------------+----------------------------------------------------------------

a_minus | 4.681452 3.519781 2.05 0.040 1.072504 20.43441

hsoshock | 3.425637 1.345284 3.14 0.002 1.586592 7.396347

------------------------------------------------------------------------------

**Rate of developing a postoperative complication**

**(Results of stepwise Cox regression analysis)**

begin with full model

p = 0.9948 >= 0.1000 removing pyloric

p = 0.9830 >= 0.1000 removing vomitting

p = 0.9794 >= 0.1000 removing contamicat

p = 0.9113 >= 0.1000 removing fever

p = 0.9149 >= 0.1000 removing prepyloric

p = 0.9229 >= 0.1000 removing oliguria

p = 0.9080 >= 0.1000 removing hsodehydration

p = 0.6847 >= 0.1000 removing hsoapd

p = 0.6656 >= 0.1000 removing a_minus

p = 0.5380 >= 0.1000 removing b_plus

p = 0.5374 >= 0.1000 removing hsoshock

p = 0.3620 >= 0.1000 removing a_plus

p = 0.2951 >= 0.1000 removing agecat2

p = 0.2012 >= 0.1000 removing durationofpaincollapsed

p = 0.1858 >= 0.1000 removing tenderness

p = 0.1906 >= 0.1000 removing bowelsounds

Poisson regression Number of obs = 96

LR chi2(4) = 69.47

Prob > chi2 = 0.0000

Log likelihood = -66.258605 Pseudo R2 = 0.3439

------------------------------------------------------------------------------

noofadvers~s | IRR Std. Err. z P>|z| [95% Conf. Interval]

-------------+----------------------------------------------------------------

bloodtrans~n | 2.381621 .6732674 3.07 0.002 1.368498 4.144778

medcondi | 2.588735 1.008879 2.44 0.015 1.206031 5.556699

ab_plus | 2.469186 1.213038 1.84 0.066 .9427225 6.467314

distension | 4.616363 1.93543 3.65 0.000 2.029692 10.49953

------------------------------------------------------------------------------

***Part C***

**Correlation matrix for the candidate prognostic predictors in patients of perforated peptic ulcer**

|  | Vomiting | Fever | Oliguria | H/o APD | H smok | H/o shock | H/o dehydration | Tenderness | Distension | Bowel sounds | Blood transfusion | Medical Condition | Age>40 |
| --- | --- | --- | --- | --- | --- | --- | --- | --- | --- | --- | --- | --- | --- |
| Fever | 0.2731 |  |  |  |  |  |  |  |  |  |  |  |  |
|  | *0.0071* |  |  |  |  |  |  |  |  |  |  |  |  |
| Oliguria | 0.3526 | 0.7100 |  |  |  |  |  |  |  |  |  |  |  |
|  | *0.0004* | *0.0000* |  |  |  |  |  |  |  |  |  |  |  |
| H/o APD | 0.0710 | 0.1423 | 0.1592 |  |  |  |  |  |  |  |  |  |  |
|  | *0.4917* | *0.1667* | *0.1213* |  |  |  |  |  |  |  |  |  |  |
| H smok | 0.0717 | -0.2176 | -0.1471 | 0.0015 |  |  |  |  |  |  |  |  |  |
|  | *0.4874* | *0.0332* | *0.1526* | *0.9886* |  |  |  |  |  |  |  |  |  |
| H/o shock | 0.4164 | 0.6900 | 0.8085 | 0.1374 | -0.1600 |  |  |  |  |  |  |  |  |
|  | *0.0000* | *0.0000* | *0.0000* | *0.1818* | *0.1194* |  |  |  |  |  |  |  |  |
| H/o dehydration | 0.5062 | 0.6476 | 0.7331 | 0.1545 | -0.1347 | 0.8592 |  |  |  |  |  |  |  |
|  | *0.0000* | *0.0000* | *0.0000* | *0.1328* | *0.1907* | *0.0000* |  |  |  |  |  |  |  |
| Tenderness | -0.0708 | -0.0716 | -0.1541 | 0.0374 | -0.0037 | -0.1634 | -0.2176 |  |  |  |  |  |  |
|  | *0.4930* | *0.4881* | *0.1339* | *0.7178* | *0.9714* | *0.1116* | *0.0332* |  |  |  |  |  |  |
| Distension | 0.5972 | 0.5073 | 0.6302 | 0.1730 | -0.0193 | 0.7005 | 0.6432 | -0.1611 |  |  |  |  |  |
|  | *0.0000* | *0.0000* | *0.0000* | *0.0920* | *0.8516* | *0.0000* | *0.0000* | *0.1169* |  |  |  |  |  |
| Bowel sounds | -0.3346 | -0.2477 | -0.3197 | -0.0190 | 0.2517 | -0.3832 | -0.4679 | -0.1160 | -0.2033 |  |  |  |  |
|  | *0.0009* | *0.0150* | *0.0015* | *0.8544* | *0.0134* | *0.0001* | *0.0000* | *0.2605* | *0.0469* |  |  |  |  |
| Bloood transfusion | 0.2375 | 0.3934 | 0.3848 | 0.1578 | -0.0130 | 0.4309 | 0.4650 | -0.1238 | 0.2859 | -0.2147 |  |  |  |
|  | *0.0198* | *0.0001* | *0.0001* | *0.1248* | *0.9002* | *0.0000* | *0.0000* | *0.2295* | *0.0048* | *0.0357* |  |  |  |
| Medical condition | 0.4912 | 0.4824 | 0.3892 | 0.1287 | -0.0719 | 0.5217 | 0.5918 | -0.1511 | 0.5281 | -0.3619 | 0.4206 |  |  |
|  | *0.0000* | *0.0000* | *0.0001* | *0.2114* | *0.4866* | *0.0000* | *0.0000* | *0.1416* | *0.0000* | *0.0003* | *0.0000* |  |  |
| Age >40 | 0.4352 | 0.3581 | 0.4123 | 0.0785 | 0.0075 | 0.3889 | 0.4026 | -0.1421 | 0.5118 | -0.3033 | 0.1956 | 0.5632 |  |
|  | *0.0000* | *0.0003* | *0.0000* | *0.4472* | *0.9420* | *0.0001* | *0.0000* | *0.1673* | *0.0000* | *0.0027* | *0.0562* | *0.0000* |  |
| Duration of pain | 0.2811 | 0.2772 | 0.3853 | 0.1699 | -0.1325 | 0.4526 | 0.4503 | -0.2137 | 0.3592 | -0.4062 | 0.2440 | 0.3343 | 0.2806 |
|  | *0.0055* | *0.0063* | *0.0001* | *0.0980* | *0.1980* | *0.0000* | *0.0000* | *0.0365* | *0.0003* | *0.0000* | *0.0166* | *0.0009* | *0.0056* |

Note: The table shows the correlation coefficients between pairs of prognostic predictors used in the study and their statistical significance. The coefficients are shown in regular font while the significance values are italicized.
